# Design of Insulation Tape Tension Control System of Transformer Winding Machine Based on Fuzzy PID

**DOI:** 10.3390/s21196512

**Published:** 2021-09-29

**Authors:** Liwei Deng, Hongfei Suo, Haonan Ren

**Affiliations:** Heilongjiang Provincial Key Laboratory of Complex Intelligent System and Integration, School of Automation, Harbin University of Science and Technology, Harbin 150080, China; 13563103295@163.com (H.S.); rhn1996@163.com (H.R.)

**Keywords:** fuzzy PID, tension control, transformer, winding machine

## Abstract

With the rapid development of science and technology as well as the comprehensive societal progress, the demand for electricity in all walks of life is also increasing. As is known to all, the mechanical structure and tension control of a transformer winding machine is the key to improving the quality of coil winding, due to coil winding being generally considered the core technology of transformer manufacturing. Aiming at the synchronous winding control problem of the conductor and insulating layer of the transformer winding machine, this paper presents a mechanical structure and tension control scheme of a new type of transformer winding machine. Based on the dynamic analysis and modeling of the mechanical structure of the winding machine, the speed control of the main speed roller by the fuzzy PID control rate is implemented initially. Combined with the actual demand of the project, the feasibility and effectiveness of the control target with different tension are verified by the simulation experiment and further compared with the traditional PID control method. The simulation results show that the proposed fuzzy PID control rate can realize the automatic and efficient winding of the transformer winding machine, showing that it is superior to the traditional PID control rate in overcoming the disturbance and controlling effect.

## 1. Introduction

In recent years, with the rapid development of domestic economy, power transmission, and distribution transformers are in noticeably booming demand in the power industry. Transformers, as the basic equipment for power transmission and transformation, are widely applied in transformer stations, converter stations, and power plants, playing a significant role in the power industry [1]. The core process of transformer manufacturing is coil winding, of which winding quality directly affects the overall performance of the transformer. The technical level of coil winding largely depends on the technical level of the winding machine [2]. Therefore, in order to manufacture high-performance transformers, the priority is to boost the development of technical level of winding machines.

The mechanical structure of the tension system and constant tension control are the core technologies of the transformer winding process. The transformer winding machine developed by several well-known companies has a high degree of automation and production efficiency, and also, the winding coil is constant in integrated tension and guaranteed in quality. The MTM Company of Canada and LAE Company of Italy have achieved the synchronous winding of conductor and insulating tape, remarkably improving the production efficiency [3]. The EFECO 800 automatic winding machine, produced by Tuboly-Astronic AG, Switzerland, has achieved fully automatic control of coil winding, automatic coil winding without manual intervention, and precise tension control of wires and insulating materials during the winding process [4]. As far as tension system control is concerned, in 2001, Mahawan et al. proposed a new electromechanical control tracking system of winding and a new online identification scheme of the general servo mechanism, which were applied to winding machine equipment, thereby assuring the constant tension control of the whole control system even under great disturbance [5]. In 2008, Wen et al. designed a tension control scheme by adding a flattening machine including an accumulator and a tensioning device, which replaced the traditional energy storage device and made it possible to vary the winding speed under a certain tension fluctuation without prejudicing the winding quality [6]. In 2010, Ponsart et al. successfully applied the observer theory to the transformer winding machine in order to realize the fault estimation in the winding process of the winding machine, meanwhile adopting the LTV model to improve the control accuracy of the web tension [7,8,9]. In 2015, Le-Bao et al. proposed a new multi-motor speed tracking and synchronization control method, using multiple motors to make the speed error and synchronization error converge to 0, and they simultaneously verified that the control scheme had robust interference to parameter changes [10]. In 2019, Quanjin et al. designed a mobile software for a wirelessly connected three-axis fiber winding machine. Through Bluetooth, the parameters such as the winding angle, length, and thickness of the three-axis fiber winding machine could be controlled in real time, so that the crux of the matter such as the complex wiring form of the fiber winding machine could be solved. In result, the experimental verification was in line with expectations [11].

As a result of the technical restriction caused by both foreign technology blockade and limited development investment, the winding machine developed in China is still semi-automatic. With the manual pedaling method, it can only wind the wire semi-automatically, inducing several downsides of low production efficiency and low transformer core technical parameters such as low window filling factor. The GRX-800 automatic winding machine developed by a company in Shandong and the F-TW100 CXL transformer winding machine produced by a company in Dongguan can both achieve the constant tension control winding of the wire, yet the synchronous winding of the insulation belt and the wire is incapable. Many scholars have conducted research on the tension control system of the winding machine. Shi Yaoyao et al., centering on the winding process of the discontinuous strip, conducted in-depth analysis and research on key technologies such as tension control, automatic continuous step, and automatic calibration in the winding process, and they designed a PID controller to realize the winding of a discontinuous strip [12]. Xue Bingren from the Harbin Institute of Technology designed an automatic winding machine control system with PLC as the control core and accordingly put forward a feasible tension control scheme to achieve constant tension control of wire winding [13]. Hongqian et al. designed an automatic winding control system for the textile industry, aiming at replacing the manual winding of broken textile thread on the empty wire barrel. However, there are still some deficiencies in the above research, such as uncooperative winding, insufficient control accuracy at high speed, and a small tension control range; therefore, the outputs cannot be directly applied to the research and development of a 35 kVA transformer winding machine [14]. Zhiyong et al. brought up a creative method for cable force distribution optimization of a cable-driven parallel robot; that is, the minimum tension of the cable was modified based on the dynamic characteristics of the system. Different controllers were adopted to conduct experiments on a two-cable one-DOF test bench. Experimental results indicate that the dynamic minimum tension control (DMTC) was superior to the traditional minimum tension method in terms of accuracy and energy consumption [15].

On account of the stated needs of enterprise for the automatic winding machine, this paper analyzes the mathematical relationship between tension control and speed through the mathematical modeling of the insulated belt structure of winding machines on the basis of research results of foreign automatic winding machines. Based on the fuzzy control theory, an innovative tension control scheme is proposed, which takes the main speed roller as the control object. By converting a single precise digital input into a large number of fuzzy sets, the robustness of tension control is exquisitely enhanced. By comparing the simulation experiment to the traditional PID scheme, the efficiency of the proposed fuzzy control rate is also detected, as well as the experimental verification carried out on the engineering inspection machine.

## 2. Mechanical Structure and Dynamics Model

**Figure 1:**
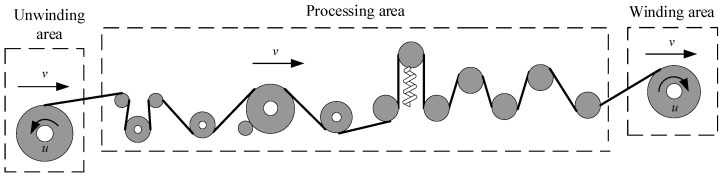
Mechanical structure of insulating tape system.

The automatic winding machine includes the automatic winding of a conductor and insulating layer. The tensile capacity of the insulation layer is limited so that it can be difficult to control. The winding tape is too easy to bend and break; hence, it becomes the core problem of the whole winding machine design and control. Taking the insulating tape system as an example, this paper studies the constant tension control algorithm. The mechanical structure of the insulating tape system is shown in Figure 1, including the unwinding area, processing area, and rewinding area. In the winding area and unwinding area, the DC motor is used as the actuator to control the production of these two parts. During the winding process, the electromagnetic brake roller, the main speed roller, and the guide roller in the processing area control the tension of the reel. Using an AC servo motor as the actuator, the speed control of the main speed roller is carried out through the speed control of the AC servo motor [16,17,18].

**Figure 2:**
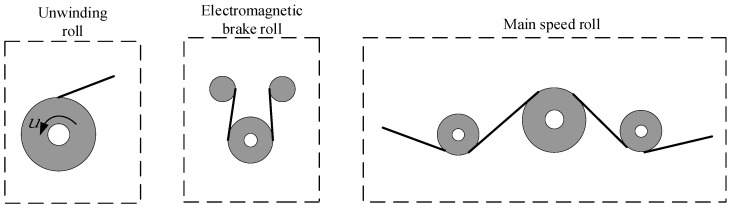
Main devices in the winding process of insulating tape.

The main devices of the transformer winding machine in the winding process are shown in Figure 2. There are three devices in total, whose characteristics are shown in Table 1. The control system can control the tension and speed of the insulation tape winding by adjusting the output of three different driven rollers, namely, the torque of the unwinding roller, the torque of the magnetic powder brake roller, and the speed of the driving roller [19].

In the first stage, the unwinding roller (driven by a torque motor) generates a reverse force to apply a pretension on the winding coil, which is set to a smaller value to reduce the influence of the time-varying radius of the unwinding coil and periodic oscillations on the preset tension. Coming to the second stage, the electromagnetic brake takes advantage of the fact that it can generate torque over a wide range without introducing significant tension interference to control tension. In the final stage, when the tension of the winding coil is restored to set the tension value, the speed of the winding coil is controlled by controlling the speed of the main speed roller, which tracks the linear speed of the winding coil and uses it as the reference speed and adjusts the speed to maintain the expected value of the tension [20,21].

According to the mechanical structure of the transformer winding machine and the winding characteristics of the insulating tape in Figure 1, a mathematical model of the whole insulating tape tension control system is established. Taking the insulated belt system as an example, mathematical modeling is carried out for winding roll, electromagnetic brake roll, main speed roll, and guide roll, respectively [22].

### 2.1. Dynamic Model of Roller

**Figure 3:**
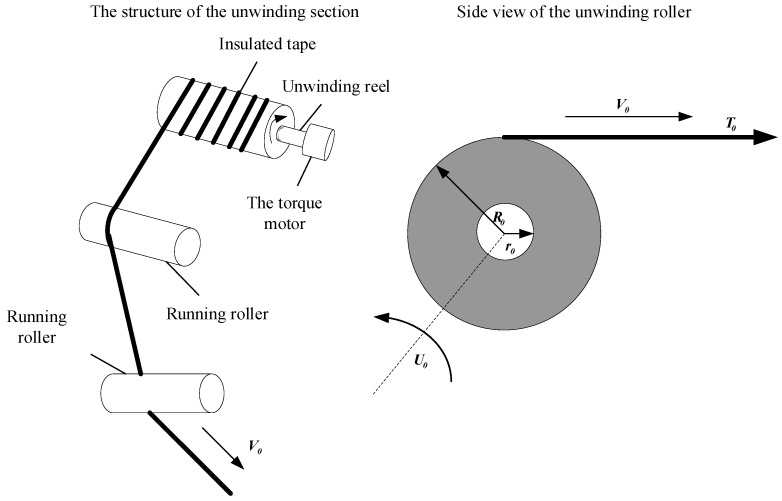
Mechanical structure of unwinding part and side view of the unwinding roller.

Figure 3 shows the mechanical structure side view of the unwinding part of the transformer winding machine and the unwinding roller. There is an insulated core arranged on the unwinding shaft which is driven by a torque motor. In the unwinding process, the relationship between coil winding tension and velocity is as follows:

(1) $$\{\begin{array}{c} T_{0}R_{0}=I\alpha +T_{n}+T_{b}\\ \frac{d}{dt}\left(J_{0}\left(t\right)w_{0}\right)=T_{0}R_{0}-n_{0}^{2}U_{0}-b\omega_{0} \end{array}$$

where $T_{0}$, $T_{n}$, and $T_{b}$ are the tension, friction torque, and load torque of the unwinding insulation tape and the motor, respectively. $R_{0}$ is the radius of the unwinding coil, I is the moment of inertia, and α is the angular acceleration during unwinding. $J_{0}\left(t\right)$ is the moment of inertia, $\omega_{0}$ is the initial angular velocity, $U_{0}$ is the input torque of the torque motor, $n_{0}$ is the mechanical transmission ratio between the torque motor and the unwinding roller, and b is the friction coefficient.

At any moment, the moment of inertia of the unwinding roller is composed of three parts:

(2) $$J_{0}\left(t\right)=J_{m}+J_{c}+J_{\omega }\left(t\right)$$

where $J_{m}$ is the moment of inertia of the torque motor, $J_{c}$ is the moment of inertia of the winding shaft, and $J_{\omega }\left(t\right)$ is the moment of inertia of the insulating belt.

Due to the real-time change of the coil diameter during the winding process of the unwinding roller, $J_{\omega }\left(t\right)$ will also change in real time. The analysis is expressed as follows:

(3) $$\{\begin{array}{c} J=\frac{1}{2}m\left(R_{2}^{2}+R_{1}^{2}\right)\\ m=\rho \pi l\left(R_{2}^{2}-R_{1}^{2}\right)\\ J_{\omega }\left(t\right)=\frac{\pi }{2}\rho W\left(R_{0}^{2}-r_{0}^{2}\right)\left(R_{0}^{2}+r_{0}^{2}\right)=\frac{\pi }{2}\rho W\left(R_{0}^{4}-r_{0}^{4}\right) \end{array}$$

where ρ is the density of the insulating strip, W is the width of the insulating strip, $R_{0}$ is the radius of the unwinding roll, and $r_{0}$ is the radius of the insulating strip core. Therefore, Equation (1) can be written as:

(4) $${\dot{J}}_{0}\omega_{0}+J_{0}{\dot{\omega }}_{0}=T_{0}R_{0}-n_{0}^{2}U_{0}-b\omega_{0}.$$

Thereinto:

(5) $$\{\begin{array}{c} {\dot{J}}_{0}=2\pi \rho WR_{0}^{3}{\dot{R}}_{0}\\ \omega_{0}=v_{0}R_{0} \end{array}.$$

Therefore, Equation (4) can be written as:

(6) $$T_{0}R_{0}=2\pi \rho Wv_{0}R_{0}^{4}{\dot{R}}_{0}+J_{0}{\dot{v}}_{0}+J_{0}v_{0}{\dot{R}}_{0}+n_{0}^{2}U_{0}+bv_{0}R_{0}.$$

The above expression illustrates the relationship between the tension $T_{0}$, the rate of change ${\dot{R}}_{0}$, and the velocity differential expression in the unwinding process [23,24].

### 2.2. Dynamic Model of Electromagnetic Brake Roller

**Figure 4:**
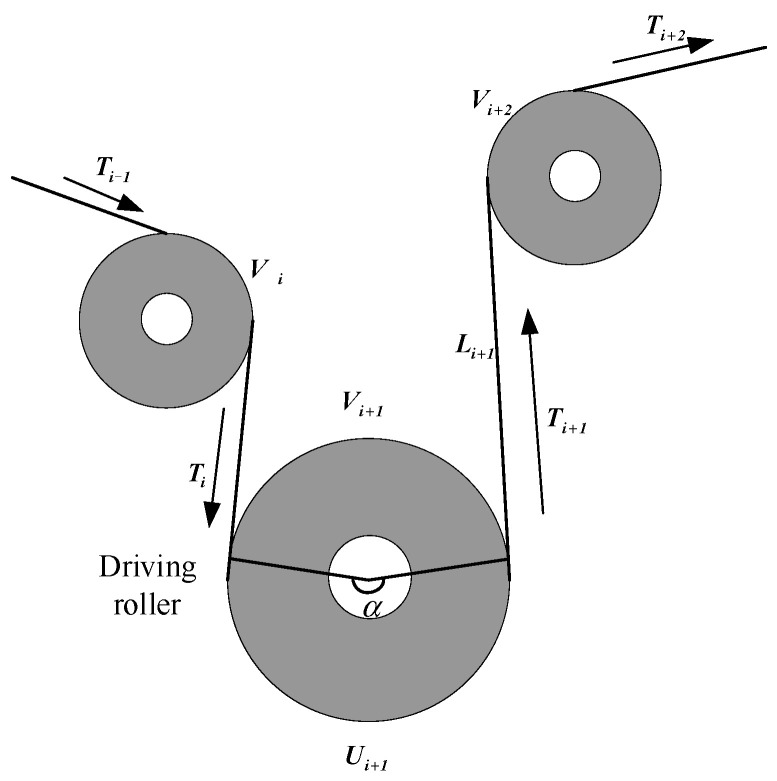
Analysis of electromagnetic brake.

The electromagnetic brake roller of the transformer winding machine, which consists of one driving roller and two driven rollers, can exert large tension on the insulating tape during winding. The analysis diagram of the electromagnetic brake is shown in Figure 4.

The relationship between the preset tension $T_{i}$ of the insulating tape and the maximum tension $T_{i}^{max}$ allowed to be applied during winding is as follows:

(7) $$\frac{T_{i}^{max}}{T_{i}}=e^{u\alpha }$$

where u is the friction coefficient between the driving roller and the insulating belt, and α is the central angle. In general, α and u are important for achieving high tension: the greater the value of α and *u*, the greater the maximum tension applied by the electromagnetic brake.

Meanwhile, the driving roller provides the main tension in the winding process. The dynamic modeling of the driving roller is shown in Equation (8):

(8) $$J_{i}\frac{d\omega_{i}}{dt}=\left(T_{i+1}-T_{i}\right)R_{i}-nU_{i}-b\omega_{i}$$

where $J_{i}$ is the rotational inertia of the driving roller, $\omega_{i}$ is the angular velocity of the driving roller, $T_{i}$ is the input tension, $T_{i+1}$ is the output tension, $R_{i}$ is the radius of the driving roller, $U_{i}$ is the input torque of the driving roller motor, n is the mechanical transmission ratio of the driving roller and the motor, and b is the friction coefficient.

The mathematical relationship between the tension and speed on the driving roller is obtained by further derivation, as shown in Equation (9):

(9) $$L_{i+1}\frac{d\left(T_{i+1}\right)}{dt}=AE\left(v_{i+2}-v_{i+1}\right)+T_{i}v_{i+1}-T_{i+1}v_{i+2}$$

where $L_{i+1}$ is the stretching amount of the insulating strip, E is the elastic modulus of the insulating strip, A is the cross-sectional area of the insulating strip, and $v_{i+1}$ and $v_{i+2}$ are the velocities before and after the insulating strip stretching, respectively.

The incremental model of the driving roller is expressed as:

(10) $$J_{i}\left({\dot{v}}_{i}^{r}+{\dot{v}}_{i}^{q}\right)=\left(T_{i+1}^{r}+T_{i+1}^{q}-T_{i}^{r}-T_{i}^{q}\right)R_{i}^{2}-nU_{i}^{r}R_{i}-b\left(\omega_{i}^{r}+\omega_{i}^{q}\right)R_{i}$$

where ${\dot{v}}_{i}^{r}$ is the reference velocity, ${\dot{v}}_{i}^{q}$ is the velocity increment, $T_{i}^{r}$ is the reference tension, $T_{i}^{q}$ is the tension increment, and $U_{i}^{r}$ is the reference torque. At equilibrium, the increment of tension and velocity could be regarded as zero.

(11) $$\left(T_{i+1}^{r}-T_{i+1}^{q}\right)R_{i}^{2}-nU_{i}^{r}R_{i}-b\omega_{i}^{r}R_{i}-J_{i}{\dot{v}}_{i}^{r}=0$$

Therefore, Equation (10) can be written as:

(12) $$J_{i}{\dot{v}}_{i}^{q}=\left(T_{i+1}^{q}-T_{i}^{q}\right)R_{i}^{2}-b\omega_{i}^{q}.$$

$T_{i+1}^{q}$ and $T_{i}^{q}$ can be expressed as Equation (13):

(13) $$T_{i+1}^{q}=\frac{J_{i}{\dot{v}}_{i}^{q}}{R_{i}^{2}}+\frac{b\omega_{i}^{q}}{R_{i}^{2}}+T_{i}^{q}.$$

The above equation shows that as the radius and torque decrease, the increment of tension also decreases, and the increment of tension is independent of the reference torque; hence, the increase in tension can be achieved without introducing interference [25,26].

### 2.3. Dynamic Model of Main Speed Roller

**Figure 5:**
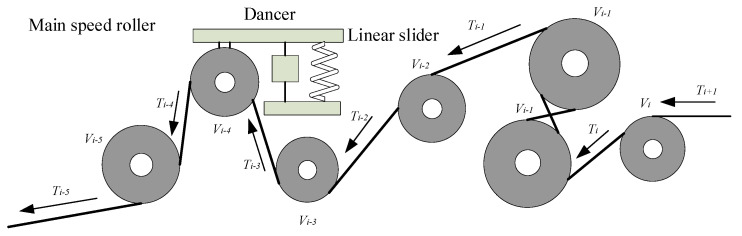
Cross-section view of the main speed roller.

The main speed roller of the transformer winding machine is driven by a servo motor to track the unwinding speed and adjust tension. Its cross-section is shown in Figure 5. The Dancer mechanism is introduced to reduce tension and velocity interference. The floating roller in Dancer can move freely on the linear slider. A cylinder installed between them increases the Dancer’s response time. In the steady state, the floating roller is in equilibrium, and the tension control is within the predetermined range. The change in speed will cause the change in tension, causing the roller deviating from the balance position, eventually resulting in the main speed roller adjusting the speed so as to make the floating roller return to the balance position. Dancer deviates from the equilibrium position and then returns to the equilibrium position to maintain stable tension [27].

The dynamic model of the main speed roller is as follows:

(14) $$L_{i+1}\frac{d\left(T_{i+1}\right)}{dt}=AE\left(v_{i+2}-v_{i+1}\right)+T_{i}v_{i+1}-T_{i+1}v_{i+2}.$$

### 2.4. Dynamic Model of Guide Roller

**Figure 6:**
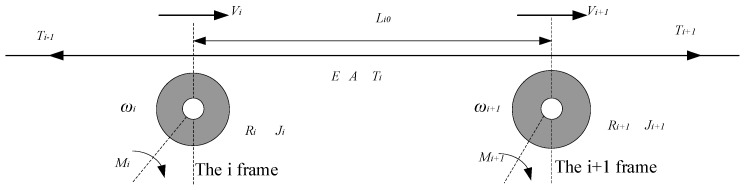
Sectional view of guide roller.

There are many idling rollers called guide rollers in the working process of the insulation belt of the transformer winding machine. Since the acceleration of the guide roller is very small in the steady-state process, the influence of the guide roller in the transmission process can be ignored. However, if the acceleration and deceleration are frequent, the movement of the guide roller will also have a great impact on the tension. The section view of the guide roller is shown in Figure 6.

In the winding process of the transformer winding machine, the tension of the insulating strip at section *i* is $T_{i}$, the linear velocity is $V_{i}$, the angular velocity is $\omega_{i}$, the length before and after stretching is $L_{i0}$ and $L_{i}$, the radius of the *i*-th roll is $R_{i}$, the moment of inertia of the roll is $J_{i}$, and the electromagnetic torque is $M_{i}$.

(15) $$T_{i}=EA_{\varepsilon i}=EA\frac{L_{i}-L_{i0}}{L_{i0}}=EA\frac{\Delta L_{i}}{L_{i0}}$$

The time interval between the two guide rollers is $t_{i}$.

(16) $$\Delta L_{i}-L_{i0}=\int_{0}^{t_{i}}\left(V_{i}-V_{i-1}\right)dt$$

Therefore, Equation (17) can be derived from Equations (15) and (16):

(17) $$T_{i}=\frac{EA}{L_{i0}}\int_{0}^{t_{i}}\left(V_{i+1}-V_{i}\right)dt=\frac{EA}{L_{i0}}\int_{0}^{t_{i}}\left(\omega_{i}R_{i+1}-\omega_{i}R_{i}\right)dt.$$

As can be seen from the above equation, when the speed of the adjacent guide rollers remains constant, the tension of the insulating belt will not be affected. If the speed of the back guide roller is less than that of the front guide roller, the tension will increase. If the speed of the back guide roller is greater than that of the front guide roller, the tension becomes smaller [28,29].

## 3. Design of Fuzzy Control System for Winding Machine

### 3.1. Fuzzy PID Control

**Figure 7:**
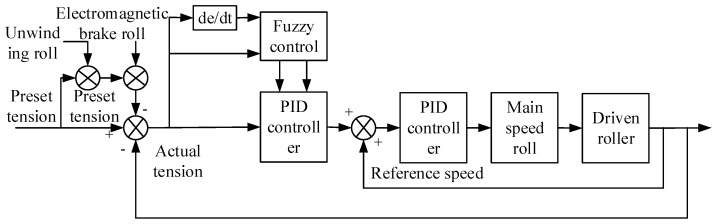
Tension control based on fuzzy PID.

In view of the synchronous winding of the conductor and the insulating layer of the transformer winding machine, the idea of fuzzy control is introduced, and a fuzzy tension control scheme is proposed, as shown in Figure 7. The preset tension is taken as the input of the transformer winding machine, and the input variable is expressed as x(k). In order to ensure the smooth start of the system, we adopt the speed and tension double closed-loop adjustment. The actual tension of the outer loop is the feedback value, and a fuzzy controller is designed for speed correction to convert the original feedback of a single precise digital quantity into a fuzzy set. The feedback value of the inner closed loop is the reference speed, and the sum of the reference speed and the output speed of the controller is the input command of the main speed roller, so that the conductor and the insulation belt can be wound synchronously [30].

The PID controller is a fuzzy logic controller, and the adaptive fuzzy controller can be described by Equation (18):

(18) $$\{\begin{array}{c} x\left(k\right)={\left[e\left(k\right)/K_{1},∆e\left(k\right)/K_{2}\right]}^{T}\\ e\left(k\right)=t\left(k\right)-t_{r}\left(k\right),∆e\left(k\right)=e\left(k\right)-e\left(k-1\right)\\ u\left(k\right)=\left[∆Kp\left(k\right),∆K_{d}\left(k\right)\right] \end{array}$$

where x(k) is the input variable, e(k) is the difference between the preset and the actual tension, ∆e(k) is the difference between the preset and the actual tension, $K_{1}$ is the scaling factor of e(k), $K_{2}$ is the scaling factor of ∆e(k), t(k) is the input tension, $t_{r}\left(k\right)$ is the reference tension, u(k) is the output variable, ∆Kp(k) is the change in the scale factor, and $∆K_{d}\left(k\right)$ is the change in the differential factor. The output u(k) has three phases: fuzzification, fuzzy rule, and defuzzification, which are explained in detail below.

### 3.2. Fuzzification

**Figure 8:**
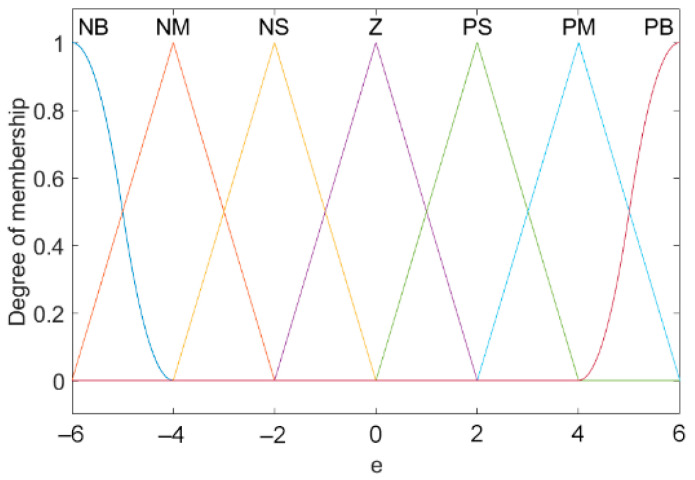
Membership function of e(k).

**Figure 9:**
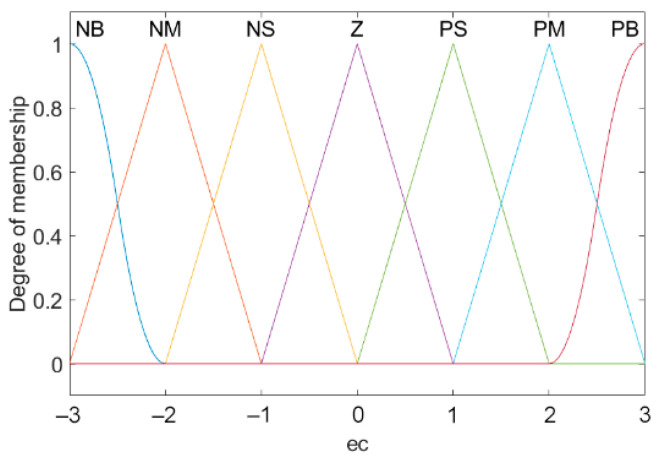
Membership function of ∆e(k).

The basic domain is the actual range of parameter changes. Refer to the actual situation to set the basic domain of e(k) to [−6, 6], and the basic domain of ∆e(k) to [−3, 3]. The quantities in the basic domain are exact values, as are the input and output of the controller. Yet the fuzzy control algorithm needs fuzzy quantity [31,32]. As a result, it is crucial to introduce fuzzification to convert the precise input quantity into fuzzy quantity. The key of fuzzification is to input it into the fuzzy set by membership function. Figure 8 and Figure 9 respectively represent the membership functions of input e(k), ∆e(k). The membership function of the output is the same as that of the input, and the value range of the output is [−5, 5].

### 3.3. Fuzzy Rule

The core content of fuzzy control is the formulation of fuzzy rules, including rule base and fuzzy reasoning [33,34]. The rule bases based on $K_{p}\left(k\right)$ and $K_{d}\left(k\right)$ are shown in Table 2 and Table 3.

### 3.4. Defuzzification

**Figure 10:**
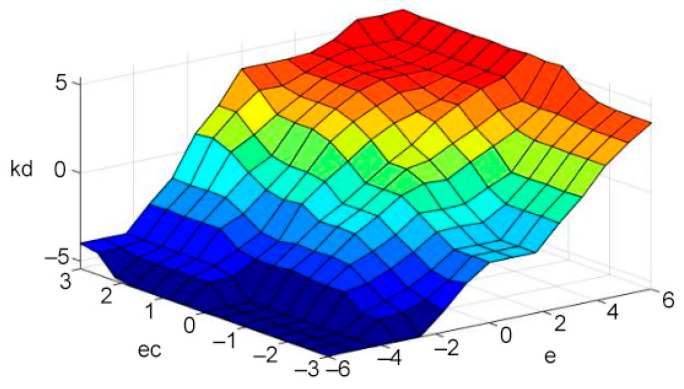
The change of $∆K_{d}\left(k\right)$.

**Figure 11:**
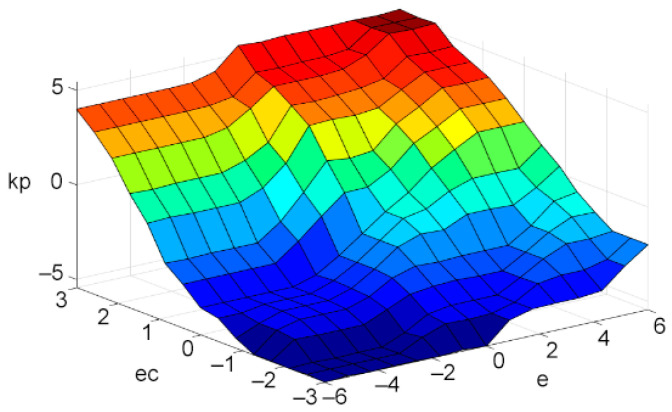
The change of $∆K_{p}\left(k\right)$.

The control quantity u obtained by fuzzy decision is a matrix, which cannot be directly applied to engineering. Therefore, u needs to be interpreted as a specific behavior in practice, that is, defuzzification operation [35,36]. At present, several of the most commonly used defuzzification methods are as follows: maximum membership degree method, gravity center method, and weighted average method [37]. In this paper, the center of gravity method is selected. The formula of the center of gravity method is shown as Equation (19) [38], and the output results of $∆K_{d}\left(k\right)$ and $∆K_{p}\left(k\right)$ are shown in Figure 10 and Figure 11.

(19) $$u\left(k\right)=K_{out}\frac{\sum u\left(u_{j}\right)u_{j}}{\sum u_{j}}$$

## 4. Simulation and Result Analysis

### 4.1. System Mechanical Parameters

The mechanical parameters of the system are shown in Table 4.

### 4.2. Analysis of Simulation Result

**Figure 12:**
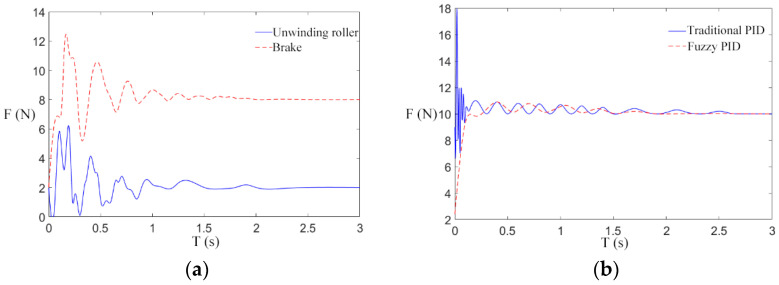
Tension response at 10 N reference tension. (**a**) Tension of unwinding roller and electromagnetic brake; (**b**) Tension of take-up roll.

**Figure 13:**
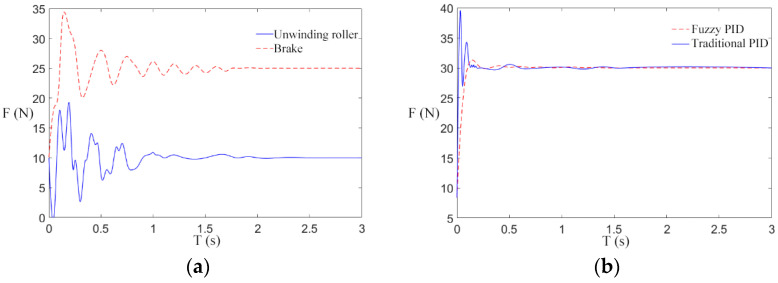
Tension response at 30 N reference tension. (**a**) Tension of unwinding roller and electromagnetic brake; (**b**) Tension of take-up roll.

**Figure 14:**
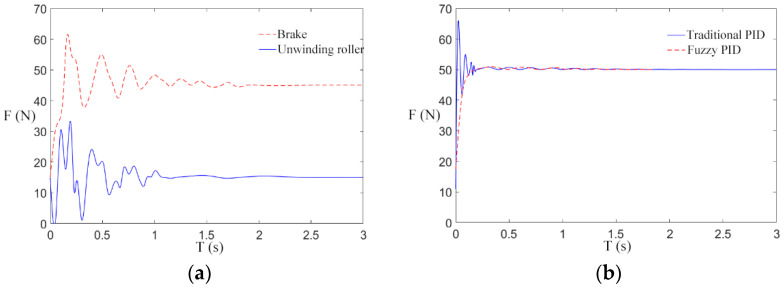
Tension response at 50 N reference tension. (**a**) Tension of unwinding roller and electromagnetic brake; (**b**) Tension of take-up roll.

We try our best to establish the simulation environment for the paper under the debugging state of the engineering prototype. The simulation parameters involved in this paper are mainly from relevant references and actual parameters that are closer to the real state. According to the set mechanical parameters, the rationality of the design of a fuzzy PID control scheme for the transformer winding machine is verified. The rewinding speed is set as 1 m/s, and the reference tension is 10 N, 30 N, and 50 N to carry out multiple groups of simulation experiments. The tension responses obtained were shown in Figure 12a, Figure 13a, and Figure 14a.

As can be seen from Figure 12a, Figure 13a, and Figure 14a, when the winding machine is just started, the tension of the unwinding roller and the electromagnetic brake roller fluctuate greatly around the reference value. There are two possible reasons for this: (1) The tension fluctuations generated by the interaction of velocities are susceptible to the variation of the time derivative of velocities. (2) The periodic swing of the unwinding roller will produce tension instability. Under the action of fuzzy control, the tension generated by the unwinding roller is small, and the tension disturbance of the magnetic brake roller is small as well. Therefore, the controller can reach the steady state faster than the traditional PID controller while diminishing the error in the steady state. The controller is designed to take a certain amount of time to track the reference tension, and the greater the reference tension, the shorter the time required.

The tension response of the rewinding roller under the traditional PID control scheme and the fuzzy PID control scheme is compared, and the reference tensions are 10 N, 30 N, and 50 N. The tension responses are shown in Figure 12b, Figure 13b and Figure 14b.

As shown in Figure 12b, Figure 13b and Figure 14b, after the winding machine is started, the rewinding roller rewinds, and the radius of the coiling material suddenly increases, resulting in the fluctuation of rewinding tension. The tension of the winding roll obtained by the traditional control scheme fluctuates within the range $\left[\begin{array}{cc} 8 & 18 \end{array}\right]$, $\left[\begin{array}{cc} 10 & 40 \end{array}\right]$, and $\left[\begin{array}{cc} 12 & 65 \end{array}\right]$, respectively, while the tension obtained by the fuzzy control rate fluctuates within the range $\left[\begin{array}{cc} 9 & 11 \end{array}\right]$, $\left[\begin{array}{cc} 29 & 32 \end{array}\right]$, and $\left[\begin{array}{cc} 48 & 51 \end{array}\right]$, respectively. It can be seen from the figure that the tension curve of the rewinding roller is more stable under the fuzzy control rate. Combined with the actual engineering, the winding radius of the transformer winding machine increases steadily in the winding process, and the fuzzy PID tension control scheme is consistent with the actual situation. The longitudinal comparison of Figure 12b, Figure 13b, and Figure 14b suggests that under the traditional control scheme, the tension curve is gradually smooth and the tension control effect is gradually better as the reference tension increases after the winding machine is started. Under the fuzzy control scheme, with the increase in the reference tension, the fluctuation range of the tension curve is consistent with the reference tension after the winding machine starts, and the tension control is stable and not affected by the reference tension.

In summary, under the action of different reference tensions, the fuzzy control scheme has a better control effect than the traditional scheme on the influence of real-time changes of the radius of the unwinding roller and the rewinding roller on tension, with smaller fluctuation and faster progress to the stable state.

**Figure 15:**
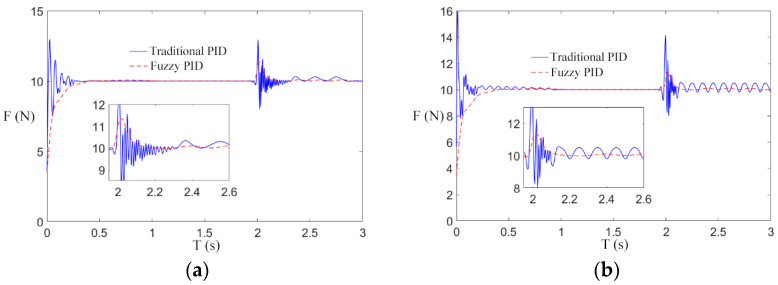
The influence of step when the reference tension is 10 N. (**a**) Situation 1; (**b**) Situation 2.

**Figure 16:**
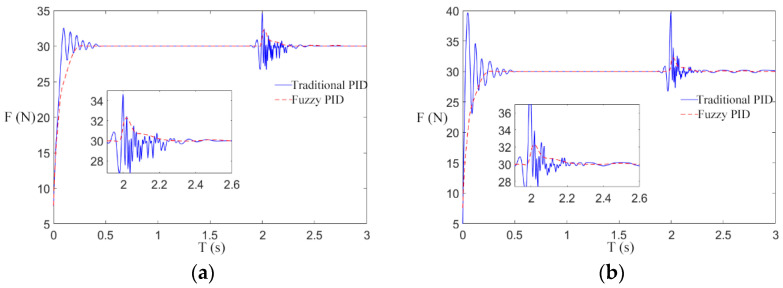
The influence of step when the reference tension is 30 N. (**a**) Situation 1; (**b**) Situation 2.

**Figure 17:**
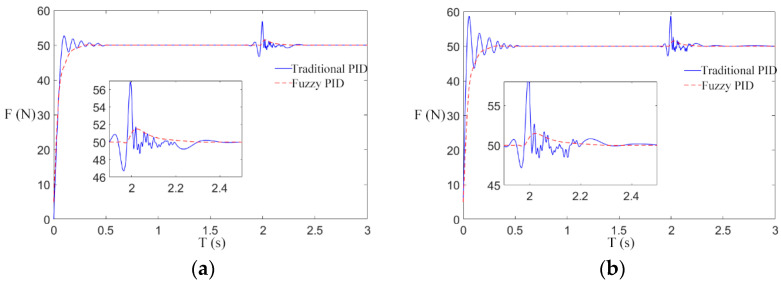
The influence of step when the reference tension is 50 N. (**a**) Situation 1; (**b**) Situation 2.

In order to further study the effectiveness of the fuzzy control scheme of a transformer winding machine in eliminating speed interference, step interference of the rewinding speed was introduced after the winding machine was started for 2 s. The parameters of speed and tension in the simulation experiment are shown in Table 5, and the simulation results are shown in Figure 15, Figure 16 and Figure 17.

In Figure 15a, Figure 16a and Figure 17a, when the winding speed is increased from 0.2 to 0.5 m/s, the sudden increase in the speed causes fluctuations in winding tension. In the traditional control scheme, when the winding machine starts for 2 s, the tension curve fluctuates greatly at the reference tension, which makes it difficult to restore stability. By using the fuzzy PID control scheme, the tension response curve is remarkably smooth at the beginning of the winding machine’s running, and it fluctuates very little 2 s after starting and soon restores stability. By comparing Figure 15a, Figure 16a and Figure 17a, it can be found that in the traditional control scheme, the tension curve of the winding machine after starting fluctuates and the amplitude decreases with the increase in the reference tension, while in the fuzzy PID control scheme, the tension curve shows no sign of obvious fluctuation.

To sum up, the simulation conclusion can be drawn: Under different reference tension, compared with the traditional control scheme, the fuzzy control scheme can reduce the interference of speed, and the experimental result shows a smooth curve, which is less affected by the reference tension.

**Figure 18:**
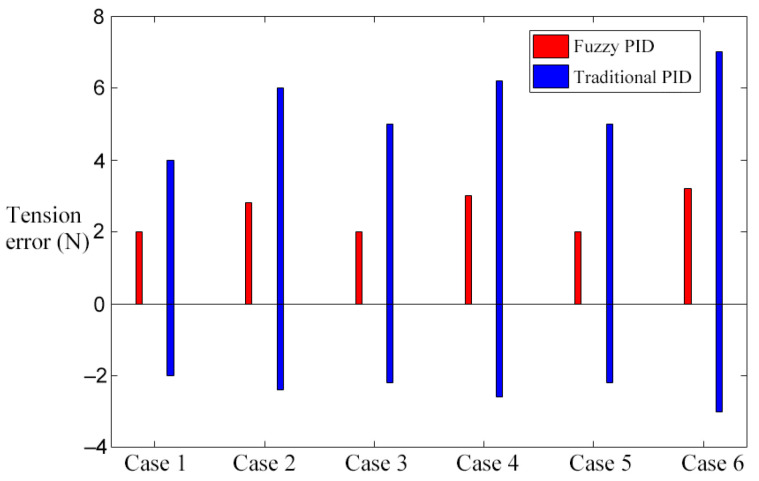
Six situations of tension fluctuation.

Figure 15b, Figure 16b and Figure 17b are the tension curves when the winding speed increases from 0.5 to 1 m/s. By comparison with Figure 15a, Figure 16a and Figure 17a, it can be seen that the tension curve of the traditional PID control scheme fluctuates significantly when the winding machine is just started and 2 s started. However, the tension of the fuzzy PID control scheme has almost no fluctuation, which further verifies that the fuzzy PID control scheme has a nice inhibitory effect on the speed interference. The peak values of the tension overshooting and downregulation in six situations are shown in Figure 18.

**Figure 19:**
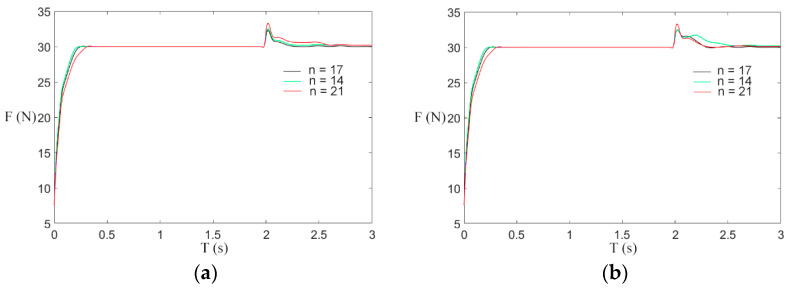
Tension response of the different number of guide rollers. (**a**) Situation 1; (**b**) Situation 2.

Combined with engineering practice, the transformer winding machine in the process of winding will adopt different process parameters and use a different number of guide rollers; therefore, this paper studies the response effect of the system to tension when the number of guide rollers varies. As shown in Figure 19, the tension response curves of guide rollers with different numbers are simulated by increasing the load mass.

As shown in Figure 19a, when the reference tension is 30 N and the winding speed increases from 0.2 to 0.5 m/s, after the winding machine starts, when the number of guide rollers n = 21, the overpunch value is 2.8 N, which is larger than the overpunch values 2.1 N and 2 N when n = 14 and n = 17, and the time of overpunch vanishing is longer when n = 21. In Figure 19b, the winding speed is increased from 0.5 to 1 m/s. When the number of guide rollers N = 21, the overpunch value is 4 N, which is larger than 3.0 N and 2.8 N when N = 14 and N = 17, but the disappearance time of overpunch is shorter. In conclusion, the number of guide rollers will affect the tension response. Since the simulation environment is more ideal than the real operating environment of the winding prototype, the simulation error in this paper is better than the real operating error of the prototype.

It can be seen from the above simulation experiment that compared with the traditional PID control, the fuzzy control scheme has a shorter response time, less influence of reference tension and speed interference, and better system stability when the number of guide rollers changes. From a comprehensive perspective, it can be regarded as a winding scheme of the transformer winding machine with finer overall performance.

## 5. Conclusions

In summary, a new tension control scheme of the transformer winding machine is presented in this paper, meanwhile realizing the synchronous winding control of the conductor and insulating layer of the transformer winding machine. In the case of constant tension, the control algorithm of the insulator belt system is studied, based on which a reasonable dynamic model is established accordingly. For the first time, the design idea of fuzzy control is integrated into the tension control system of an insulation belt, thus turning the precise speed control of the main speed roller into reality by using a fuzzy control rate. Through the simulation experiment, the comparison experiment with the traditional control scheme is presented in the form of curves. The experimental results suggest that the fuzzy PID tension control system can reach the stable state faster under different constant tension conditions. In addition, the proposed fuzzy PID control scheme compared with the traditional PID control scheme can restrain velocity disturbance more effectively for introducing the disturbance velocity step and modifying the number of guide rollers, thereby stabilizing the system when different reference tensions fluctuate less, which verifies the feasibility and superiority of the proposed scheme.

Through the unremitting efforts of the team, we have completed the simulation work in general, although there are still some remaining issues: due to professional limitations, we have not designed the transformer winding machine automatic shear system and automatic spray glue control system. In addition during the winding process of the transformer winding machine, how to control the high-speed flexible winding of wire generation is still a key point that requires further improvement. In the future, we will conduct more experiments so as to apply the fuzzy-PID tension winding technology to an actual winding system.

## Figures and Tables

**Table 1:** Characteristics of three devices.

Device	Characteristic
Unwinding roller	Apply small tension; Minimize tension deviations due to changes in velocity acceleration, radius, and inertia.
Electromagnetic brake roller	Apply high tension; Prevent the introduction of greater tension interference.
Main speed roller	Adjust the tension to get the target tension;
	Adjust the speed and track the coil speed.

**Table 2:** Rule base of ∆Kp(k).

∆*e*\*e*	NB	NM	NS	ZO	PS	PM	PB
NB	NB	NB	NB	NB	NM	NM	NS
NM	NB	NB	NM	NM	NM	NS	NS
NS	NB	NM	NM	NS	NS	NS	ZO
ZO	NM	NM	NS	NS	ZO	PS	PS
PS	NS	NS	ZO	PS	PS	PM	PM
PM	PS	PS	PM	PM	PM	PB	PB
PB	PM	PM	PM	PB	PB	PB	PB

**Table 3:** Rule base of $∆K_{d}\left(k\right)$.

∆*e*\*e*	NB	NM	NS	ZO	PS	PM	PB
NB	NB	NB	NB	NB	NB	NB	NM
NM	NB	NB	NB	NB	NM	NM	NM
NS	NB	NM	NM	NS	NS	ZO	ZO
ZO	NS	NS	ZO	ZO	PS	PS	PM
PS	NS	NS	ZO	PS	PM	PM	PM
PM	PS	PS	PS	PM	PM	PM	PB
PB	PM	PM	PB	PB	PB	PB	PB

**Table 4:** System mechanical parameters.

Parameter	Device	Numerical Value
$J_{1}$	The moment of inertia of the unwinding roller	$0.09\;{\mathrm{kgm}}^{2}$
$R_{1}$	The radius of the unwinding roller	0.08 m
$J_{2}$	The moment of inertia of the electromagnetic brake	$0.002\;{\mathrm{kgm}}^{2}$
$R_{1}$	The radius of the electromagnetic brake	0.06 m
$J_{3}$	The moment of inertia of the guide roller	$0.00005\;{\mathrm{kgm}}^{2}$
$R_{i}$	The radius of the guide roller	0.025 m
$L_{i}$	Span	0.5 m
*b*	Friction coefficient	0.0015
*L*	Unwinding length	0.02 m

**Table 5:** Speed and tension parameters.

Label	Reference Tension (N)	Initial Velocity (m/s)	Final Velocity (m/s)
Figure 15a	10	0.2	0.5
Figure 15b	10	0.5	1
Figure 16a	30	0.2	0.5
Figure 16b	30	0.5	1
Figure 16a	50	0.2	0.5
Figure 16b	50	0.5	1

## Data Availability

This study did not report any data.
